# Optimising Treatment Expectations Using a Video‐Based Intervention in Orthopaedic Surgery: Results From a Randomised Controlled Trial

**DOI:** 10.1002/ejp.70169

**Published:** 2025-11-15

**Authors:** Simon F. Zerth, Christian Volberg, Ann‐Kristin Schubert, Vanessa Ketter, Monika Sadlonova, Frank Euteneuer, Winfried Rief, Stefan Salzmann

**Affiliations:** ^1^ Department of Psychology, Clinical Psychology and Psychotherapy Philipps University of Marburg Marburg Germany; ^2^ Philipps University of Marburg, Department of Anesthesiology and Intensive Care Medicine University Hospital Marburg Marburg Germany; ^3^ Research Group Medical Ethics Philipps University of Marburg Marburg Germany; ^4^ Department of Orthopedics and Trauma Surgery Philipps University of Marburg, University Hospital Marburg Marburg Germany; ^5^ Department of Psychosomatic Medicine and Psychotherapy University Medical Center Goettingen Goettingen Germany; ^6^ Department of Cardiovascular and Thoracic Surgery University Medical Center Goettingen Goettingen Germany; ^7^ Department of Geriatrics University Medical Center Goettingen Goettingen Germany; ^8^ German Center for Cardiovascular Research (DZHK), Partner Site Lower Saxony Goettingen Germany; ^9^ Department of Psychology Vinzenz Pallotti University Vallendar Germany; ^10^ Health and Medical University, Medical Psychology Erfurt Germany; ^11^ Division of Clinical Psychology and Psychotherapy Vinzenz Pallotti University Vallendar Germany

**Keywords:** acute postsurgical pain, expectation violation, expectations, postoperative pain, psychological intervention, surgery

## Abstract

**Background:**

Psychological interventions designed to optimise patients' treatment expectations have proven effective in surgical populations. Since these interventions are often resource‐intensive, their clinical application is limited. We aimed to optimise treatment expectations in patients undergoing elective orthopaedic surgery with a brief video‐based expectation‐focused intervention. Additionally, the role of violated expectations was investigated exploratively.

**Methods:**

In a three‐arm randomised clinical trial, participants (*N* = 125) scheduled to undergo elective orthopaedic surgery received either an expectation‐focused video intervention aiming at fostering realistically positive treatment expectations, an active control video or standard medical care. The primary outcome was pain intensity. Measurements were taken at baseline as well as on postoperative days one and seven.

**Results:**

The intervention group reported increased treatment expectations compared to the standard medical care and the control group. No significant intervention effect on pain intensity was found. Patients indicating negative postsurgical expectation violation (feeling worse than expected) reported higher pain intensity, regardless of the study condition. No differences in pain intensity were found between patients, indicating positive or no expectation violation (feeling better or exactly as expected respectively).

**Conclusions:**

Patients' treatment expectations can be optimised with a brief video‐based intervention. However, the clinical relevance of this effect may be questionable. Postoperative pain intensity differed by expectation violation profiles. Avoiding a negative expectation violation might be a promising approach for expectation‐focused interventions in surgical populations.

**Significance:**

Our findings suggest that treatment expectations can be improved through a brief video‐based intervention, though this did not translate into improved postoperative outcomes. These results underscore both the potential and the limitations of very brief expectation‐focused approaches and can inform the refinement of such interventions.

**Trial Registration:**

German ClinicalTrials.gov identifier: www.drks.de, ID: DRKS00031516

## 1 Introduction

Despite continuous improvement in analgesic pain management approaches (Joshi et al. 2017), poorly managed acute postsurgical pain (APSP) is a common phenomenon, with studies estimating a prevalence of up to 80% (Gan 2017; Small and Laycock 2020; Sommer et al. 2008). Epidemiological data suggest that the intensity of APSP generally reaches moderate to severe levels, which continue to rise for a substantial part of patients in the following days (Giusti et al. 2022). Since APSP is associated with prolonged hospitalisation and is known to be a robust predictor of chronic postoperative pain (Beloeil and Sulpice 2016), it is imperative to ameliorate perioperative pain management. As many patients do not profit sufficiently from standard perioperative pain treatment, precision pain medicine approaches (Chadwick et al. 2021) are needed to further target modifiable psychological factors influencing APSP, to improve postoperative outcomes.

A growing body of evidence suggests that there are several psychological risk and resilience factors predicting the (non)occurrence of APSP (Riecke et al. 2023; Sobol‐Kwapinska et al. 2016; Volberg et al. 2024). Patients' treatment expectations, for example, which represent a central mechanism driving the placebo effect (Petrie and Rief 2019), have been shown to be associated with APSP (Sobol‐Kwapinska et al. 2016; Willingham et al. 2021). Moreover, expectation‐focused psychological interventions have yielded promising results in patients with medical conditions (Kube et al. 2018) and in surgical populations (e.g., Rief et al. 2017). For instance, in a randomised controlled trial by Benson et al. (2019), patients who received a brief verbally delivered expectation optimisation intervention exhibited less APSP after breast surgery.

One unfavourable aspect of expectation‐focused interventions that have been investigated is that they are reliant on hospital staff and therefore are cost‐intensive (Kube et al. 2018). This limits transferability into clinical practice since, even without delivering additional psychological interventions, hospitals often face understaffing challenges (Lasater et al. 2021). Further, as of yet, particularly “narrow” surgical populations have been addressed in studies investigating expectation‐focused interventions, such as patients undergoing heart surgery (Rief et al. 2017) or surgery for breast cancer (Benson et al. 2019), limiting generalisability to more common (but not less painful) surgeries, for example, fractures (Friesgaard et al. 2016).

Considering that patients’ treatment expectations may not align with clinical reality, it is reasonable to suggest that a violation of these expectations might influence patient‐reported outcomes. Therefore, another potentially relevant concept concerns the violation of treatment expectations (e.g., feeling worse than expected), which has gained little attention in behavioural medicine thus far. A recent study indicates associations of violated expectations and patient‐reported outcomes in patients undergoing hip surgery (Factor et al. 2023).

In light of these challenges, the present study aims to assess the effect of a brief video‐based expectation‐focused psychological intervention on APSP in patients undergoing surgery for a fracture of the outer extremities. To evaluate the specificity of the intervention, we included an active control group with a similar ‘dose’, as well as a standard medical care (SMC) condition. Additionally, we investigate whether violated expectations, operationalised through patients' subjective reports regarding the perceived match between preoperative expectations and their postoperative state, play a role in postoperative pain.

## 2 Methods

### 2.1 Patient Enrolment

The study was approved by the local institutional review board (Philipps University of Marburg, Department of Human Medicine Ethics Committee, no. 23–27 BO), was preregistered (German Clinical Trials Register, www.drks.de, ID: DRKS00031516) and followed the revised declaration of Helsinki. All patients gave verbal and written informed consent prior to their participation. Recruitment took place from 2 May 2023 to 26 April 2024. Inclusion criteria were a minimum age of 18 years; scheduled elective surgery (osteosynthesis) for a fracture of the upper or lower extremities within the next 3 days; ability to consent; willingness to participate, as well as a sufficient command of the German language. Patients presenting with additional tumour‐related pain and/or fractures that required multiple surgeries were excluded from study participation. Tumour‐related pain was excluded due to its complex and unique pathology (Falk and Dickenson 2014), whereas fractures requiring multiple surgeries were excluded as this was not feasible within the study design. An a priori power analysis using G‐Power (v. 3.1.9.6., (Faul et al. 2007)) indicated a required sample size of *N* = 117 to detect a small‐to‐medium intervention effect of *f* = 0.15 via mixed analysis of variance with *α* = 0.05 and *β* = 0.90.

### 2.2 Study Design

This study was a prospective randomised controlled trial conducted in a routine perioperative setting at the Department of Anesthesiology and Intensive Care Medicine and the Center for Orthopaedics and Trauma Surgery, University Hospital of Marburg, in collaboration with the Division of Clinical Psychology, Philipps University of Marburg. We hypothesised that a brief expectation‐focused video‐based intervention (1) optimises treatment expectations and (2) leads to decreased APSP. Exploratively, we investigated whether violated treatment expectations influenced postoperative outcomes.

### 2.3 Procedure

Potential participants were approached and asked to participate by members of the study team while they waited for their pretreatment consultation at the anaesthesia clinic. Baseline assessment took place between approximately 1 and 3 days prior to surgery in person, including demographic data, pain intensity, pain duration and treatment expectations. After the baseline assessment, patients were randomised to either an expectation optimisation group, an active control group or standard medical care (see 2.4). Participants allocated to intervention and control groups subsequently watched the intervention or control video in a treatment room respectively. The manipulation check took place immediately afterwards. Postoperative measures, postoperative pain intensity and treatment expectations (see 2.6), were collected at postoperative day 1 (POD 1) and postoperative day 7 (POD 7), either in person or via telephone. Perioperative analgesic intake was recorded (during surgery, at the post‐anaesthesia care unit (PACU), at POD 1, and at POD 7). Patients were additionally asked about their analgesic consumption during POD 2–POD 6. Analgesic intake was converted into morphine equivalents (MEQ) (Dinges et al. 2022). General anaesthesia as well as peripheral and neuraxial regional anaesthesia were performed according to local standard operating procedures. The standard basic postoperative analgesia protocol included ibuprofen and metamizole or paracetamol as basic analgesia. For rescue analgesia, patients received morphine or piritramide on demand, following local standards for postoperative pain therapy.

### 2.4 Intervention and Control Groups

#### 2.4.1 Expectation‐Focused Intervention

The intervention consisted of an animated video (approx. 8.30 min in duration) with a professional voice‐over that explicitly addressed patients' expectations and aimed at fostering realistically positive perioperative expectations towards the surgical procedure and postoperative pain. Specifically, the information delivered by the video‐based intervention was compiled to enhance treatment benefit, process, and personal control expectations (e.g., how to cope with APSP), while also decreasing expectations regarding adverse events (Laferton et al. 2017). One example of a statement addressing process expectations concerns information about the routineness and frequency of this type of surgery. Further, the intervention video specifically informed participants about the ‘power of expectations’, for example, by explaining the effect of positive expectations on brain areas responsible for pain modulation. Additionally, the video contained a brief imagination sequence, where participants were instructed to imagine what they would be able to do again after they successfully underwent surgery (e.g., recreational activities such as gardening or sports). Table 1 illustrates the key topics of the intervention.

**TABLE 1:** Summary of key intervention topics.

Topic	Example
Background information on fracture surgery	‘Around 15 million surgical procedures are performed in Germany every year. Various types of surgeries on bones and joints, such as the arms and legs, are among the top 20 most frequently performed procedures. Our doctors from the trauma surgery and anaesthesia departments are therefore highly experienced, particularly regarding bone fracture surgery’.
Information on pain and how it can be dealt with	‘It is important to understand that pain is not inherently a bad thing, but rather a protective function that warns the body of potential injury. In essence, it is very useful, even if it is uncomfortable’.
The role of psychological factors in medical treatments	‘Positive expectations can influence how pain is processed and perceived. An authentic belief in the effectiveness of a treatment—such as pain relief during surgery—can, by itself, enhance the treatment's success. You can think of it as the body's own pharmacy, releasing messenger substances like the body's natural pain‐relieving opioids’.
Reflecting on participants' own expectations	‘Perhaps you can find an inner image that gives you strength and reflects a realistic and positive attitude. It might be a picture of yourself enjoying a hobby you are looking forward to returning to, or of being in a place where you feel relaxed and look forward to the surgery with calmness’.

#### 2.4.2 Active Control Group

The development of the active control comparator condition was guided by the recommendations for the development, implementation, and reporting of control interventions in efficacy and mechanistic trials of physical, psychological and self‐management therapies (CoPPS statement; Hohenschurz‐Schmidt et al. 2023). The control group was provided with a video identical in its features (i.e., duration, animation style, speaker voice and tone), apart from the content. The control video focused on the topic of nutrition and the role of nutrients in bone composition and healing, while specifically not addressing expectations. Like the intervention condition, the control video also contained a brief imagination sequence where participants were asked to think of the role of nutrition in their lives. A translation of both video scripts can be found as Supporting Information (Appendix S1), as well as images of both videos to illustrate the animation style (Appendix S2).

Standard medical care entailed the standard of care, that is, a preoperative anaesthesia consultation with an anaesthesiologist.

### 2.5 Randomisation and Blinding

In this three‐group trial, we used stratified permuted block randomisation. Group allocation was randomly assigned (1:1:1) using an online tool (Sealed Envelope) with block sizes 6 and 9. Since research suggests that younger age might be associated with higher ratings of acute postoperative pain (Coppes et al. 2020; Polanco‐García et al. 2022; Riecke et al. 2023; Yang et al. 2019), age (< 65 years; 65 years and older) was chosen as a stratification variable. An age of 65 years and older was used as a cut‐off, as this age group tends to report less postoperative pain (Ip et al. 2009) and requires fewer postoperative analgesics (Chae et al. 2019). Randomisation was conducted by a study team member who was not actively involved in study procedures. Numbered opaque envelopes, which were opened consecutively, were used for allocating participants to the study groups. Block size was not known to the research team members who were opening the envelopes to prevent the anticipation of the study conditions.

### 2.6 Outcome Variables

The predefined primary outcome variable was pain intensity at rest, measured with an 11‐point numerical rating scale (NRS). The NRS represents a valid, reliable, and commonly used instrument to assess pain intensity (Karcioglu et al. 2018). Due to the heterogeneity of fractures and in order to standardise the postoperative pain assessment, pain at rest was chosen over movement‐evoked pain.

Treatment expectations were assessed with the Treatment Expectation Questionnaire (TEX‐Q, Shedden‐Mora et al. 2023), with which patients' treatment expectations regarding any treatment can be measured multidimensionally. The TEX‐Q is based on the integrative model of expectations by Laferton et al. (2017) and therefore measures expectations not one‐dimensionally but comprises six subscales measuring benefit, positive impact, negative impact, adverse events, process and behavioural control expectations respectively (Alberts et al. 2020). The total score and subscales exhibit good internal consistency (α = 0.71–0.92), and moderate to high retest reliability (*r* = 0.39–0.76). Moreover, the TEX‐Q shows divergent validity with regard to measures of generalised expectations (*r* < 0.32) (Shedden‐Mora et al. 2023).

To assess the degree of expectation violation regarding pain intensity (the deviation between expected and experienced pain), one item was created by the study team. Participants were asked to rate the extent to which their expectations differed from their current condition (‘in terms of my pain, my expectations differ from my current condition …’) on an 11‐point NRS from 0 (‘not at all’) to 10 (‘completely’), and to indicate whether they were feeling better than expected, worse than expected or exactly as they expected.

### 2.7 Data Analysis

Data distributions were inspected and screened for extreme outliers (i.e., values exceeding three interquartile ranges below quartile 1 or above quartile 3). Continuous variables are reported as means with standard deviations, and categorical variables as numbers with percentages. We employed constrained longitudinal data analysis (cLDA, Coffman et al. 2016; Liu et al. 2009) with linear mixed models to examine differences in changes in expectations (TEX‐Q sum scores) from pre‐ to post‐intervention (manipulation check), and changes in pain intensity from baseline to POD 1 and POD 7 between the three groups respectively. Post‐intervention treatment expectations (prior to surgery) for SMC were assumed to match baseline values, since no intervention took place. This reflects stable expectations in the SMC group, enabling comparison with active groups. Constrained linear mixed models were specified to incorporate baseline adjustment, with group means constrained to be equal at baseline (Coffman et al. 2016; Liu et al. 2009). Time and the interaction betweefferences in changes in expectations (TEX‐Q sum scores) from pre‐ to post‐intervention (manipulation check), and changes in pain intensity from baseline to POD 1 and POD 7 between the three groups respectively. Post‐intervention treatment expectations (prior to surgery) for SMC were assumed to match baseline values, since no intervention took place. This reflects stable expectations in the SMC group, enabling comparison with active groups. Constrained linear mixed models were specified to incorporate baseline adjustment, with group means constrained to be equal at baseline (Coffman et al. 2016; Liu et al. 2009). Time and the interaction between time and treatment were included as fixed factors for our main analysis, while a random intercept was specified at the patient level. Analgesic intake converted into MEQ was added as a covariate in our main analysis. The analysis was conducted according to the intention‐to‐treat principle, utilising maximum likelihood estimation to account for missing data and patient attrition. Exploratively, two linear regressions (for POD 1 and POD 7 respectively) were performed to test the underlying assumption that preoperative treatment expectations (pre‐ and post‐intervention) relate to acute postoperative pain intensity. Further, to test whether patients who differed in expectation violation differ in postoperative pain intensity, patients were divided into three groups (positive expectation violation, i.e., feeling better than expected; negative expectation violation, i.e., feeling worse than expected; no expectation violation). This served as a proxy measure of expectation violation. Two independent two‐way analyses of variance were computed with pain intensity (POD 1 and POD 7 respectively) as the dependent variable and exhibited expectation violation regarding pain intensity as well as treatment group as fixed factors.

Statistical analyses were run using JASP version 0.19 and SPSS version 30.

## 3 Results

**FIGURE 1:**
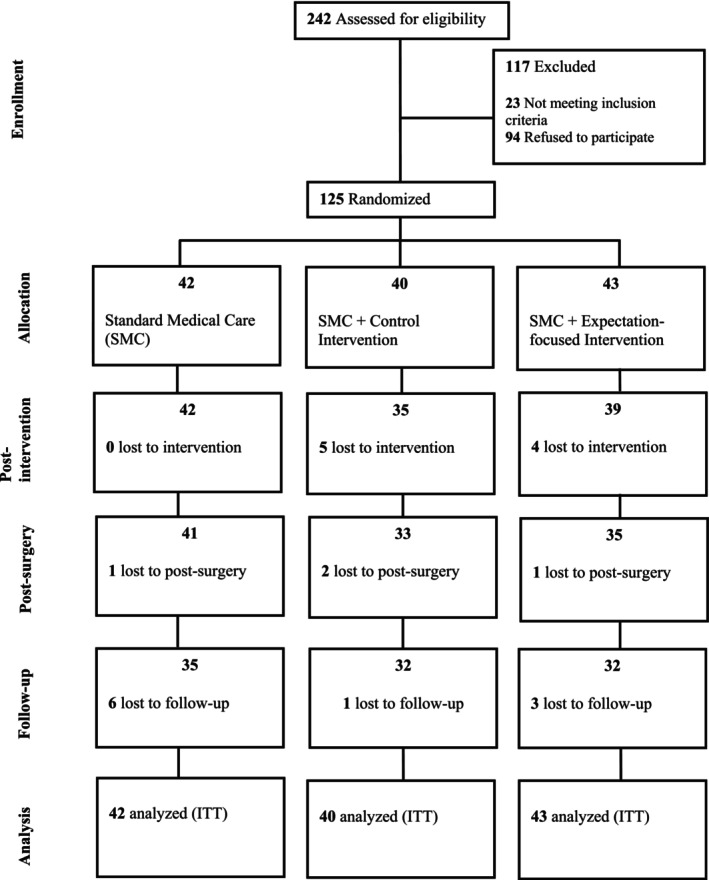
Flow of participants through each stage of the trial. SMC, standard medical care; ITT, intention‐to‐treat.

Overall, *N* = 242 patients were screened and approached for participation. In total, 125 participants were randomised into either SMC, control or intervention group. Since we followed the intention‐to‐treat principle, data of *N* = 125 participants were analysed (see Figure 1). Three extreme outliers were detected for POD 7 pain intensity; however, since these values were plausible, no participants were excluded from the analysis (for a sensitivity analysis with these outliers excluded, see supporting material S3).

### 3.1 Baseline Characteristics

One hundred twenty‐five individuals with a fracture of the outer extremities (mean age *M* = 46.60 ± 17.30 years, range: 18–81, 52% female) were analysed. Baseline characteristics are presented in Table 2.

**TABLE 2:** Baseline characteristics of the randomised sample (*N* = 125).

Variable	Intervention group (*n* = 43)	Control group (*n* = 40)	SMC group (*n* = 42)
Age (y)^a^	47.74 ± 18.40	45.83 ± 16.41	46.17 ± 17.33
Gender, *n* (% female)	26 (60.47)	20 (50.00)	19 (45.24)
Education, *n* (% high school)	23 (54.76)	19 (47.50)	26 (61.90)
Marital status, *n* (% partnered)	21 (48.84)	21 (52.50)	20 (47.62)
Setting, *n* (% inpatient)	37 (90.24)	29 (76.32)	30 (73.17)
Fracture location, *n* (% upper extremities)	28 (65.1)	28 (70.0)	27 (64.3)
Anaesthesia, *n* (% general anaesthesia)	20 (46.5)	16 (40.0)	12 (28.6)
Pain intensity^a^, ^b^ [0–10]	4.17 ± 2.55	3.51 ± 2.36	3.43 ± 2.34
Pre‐existing pain ≥ 30 days, *n* (% yes)	0 (0)	1 (2.50)	2 (4.76)
Treatment expectations^a^ [0–10]	7.73 ± 1.11	7.87 ± 1.19	7.89 ± 1.27

^*a*^ Values are presented as means (± standard deviation); y, years; d, days.

^b^ Mean pain intensity during the previous 7 days, rated on an 11‐point numerical rating scale.

### 3.2 Treatment Expectations

**FIGURE 2:**
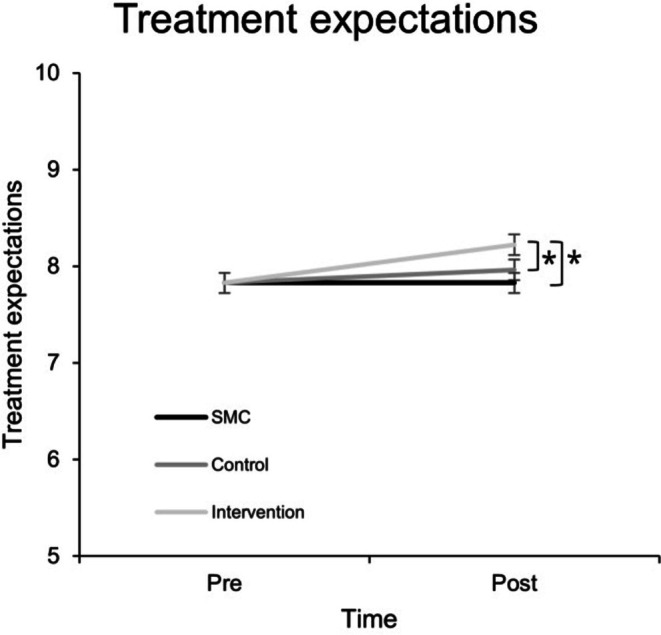
Treatment expectations at pre‐ and post‐intervention time points. Treatment expectations as measured with TEX‐Q (overall mean score); error bars represent standard error of the mean; Pre, pre‐intervention; Post, post‐intervention (approx. 10 min later). SMC, standard medical care. Pre‐intervention group means are constrained to be equal.

Constrained longitudinal data analysis was employed to check for differences in changes in treatment expectations (TEX‐Q sum scores) from pre‐ to post‐intervention (manipulation check). Ameliorated (increased) treatment expectations in the intervention group compared to SMC and the active control condition were confirmed by a significant interaction effect between time and treatment group (*F*(2,114) = 4.776, *p* = 0.010). Additionally, a significant main effect of time was observed (*F*(1, 116.260) = 10.265, *p* = 0.002), indicating that, across all conditions, treatment expectations changed over time, see Figure 2. Pairwise comparisons indicated significantly more positive treatment expectations in the intervention group compared to SMC (*M*_Δ_ = 0.391 (*p* < 0.001, 95% CI [0.189, 0.592], *d* = 0.07)), and compared to the control group (*M*_Δ_ = 0.262 (*p* = 0.049, 95% CI [0.001, 0.523], *d* = 0.05)) after the manipulation, with very small effect sizes indicating clinically negligible effects.

### 3.3 Pain Intensity

**FIGURE 3:**
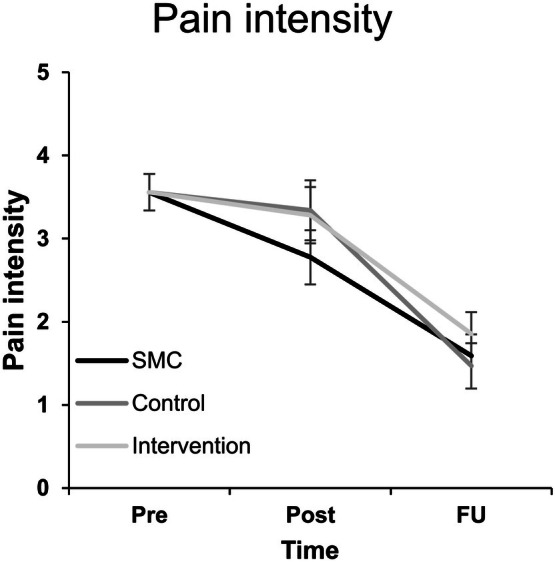
Pain scores at pre, post, and follow‐up. Pain intensity as measured with an 11‐point numerical rating scale; error bars represent standard error of the mean; Pre, pre‐intervention; Post, postoperative day 1; FU, postoperative day 7. SMC, standard medical care. Pre‐intervention group means are constrained to be equal.

Constrained longitudinal data analysis was used to examine potential differences in changes in pain intensity from baseline to post‐ (POD 1) and follow‐up (POD 7) measurements. A significant effect of time was found (*F*(2,107.99) = 69.40, *p* < 0.001). However, no significant interaction effect between time and treatment group could be detected for the primary outcome pain intensity (*F*(4,105.65) = 0.891, *p* = 0.472). Adjusted means (± SE) for POD 1 pain intensity were 2.80 ± 0.33 (SMC), 3.37 ± 0.36 (control), and 3.31 ± 0.34 (intervention). Neither the intervention condition nor the active control condition differed from SMC (intervention vs. SMC *M*_Δ_ = 0.51, 95% CI [−0.41, 1.43], *t*(108.9) = 1.09, *p* = 0.278; control vs. SMC *M*_Δ_ = 0.57, 95% CI [−0.39, 1.52], *t*(109.6) = 1.18, *p* = 0.242). For POD 7, the adjusted means were 1.63 ± 0.26 (SMC), 1.51 ± 0.27 (control), and 1.89 ± 0.27 (intervention). Again, no between‐group differences were detected (all *p* > 0.30); see Figure 3.

We conducted an additional constrained longitudinal data analysis, including anaesthesia type (general vs. regional anaesthesia) as a fixed factor to examine its potential influence on pain intensity. Neither a significant main effect of anaesthesia (*F*(1, 109.54) = 0.45, *p* = 0.505), nor a significant three‐way interaction between group, time, and anaesthesia (*F*(2, 98.89) = 0.96, *p* = 0.388) could be found.

### 3.4 Exploratory Analyses

#### 3.4.1 Associations of Preoperative Treatment Expectations With Postoperative Pain Intensity

Linear regression was employed to test whether preoperative treatment expectations (pre‐ and post‐intervention) significantly predicted pain intensity (for POD 1 and POD 7 respectively). For POD 1, a significant regression model (*F*(2,109) = 6.104, *p* = 0.003, *R* ^2^ = 0.101) indicated that pre‐intervention preoperative treatment expectations predicted postoperative pain intensity (β = −0.501, *p* = 0.004), whereas post‐intervention expectations did not (*p* = 0.139). For POD 7, the regression model was not significant (F(2, 99) = 1.371, *p* = 0.259).

We conducted further exploratory analyses to examine potential differential associations between specific expectation domains (expected benefit, expected positive impact, expected adverse events, expected negative impact, process expectations, and behavioural control expectations) and postoperative pain intensity. Since neither the initial regression model for POD 7 nor post‐intervention expectations for POD 1 yielded significant results, an additional linear regression model was fitted with the aforementioned expectation domains measured at baseline (pre‐intervention) and POD 1 pain intensity as the dependent variable. A significant regression model (F(6, 105) = 2.186, *p* = 0.050, *R* ^2^ = 0.111) indicated that only negative impact expectations were a significant predictor of acute postoperative pain intensity (β = −0.253, *p* = 0.024).

#### 3.4.2 Differences in Postoperative Pain Intensity by Expectation Violation

**FIGURE 4:**
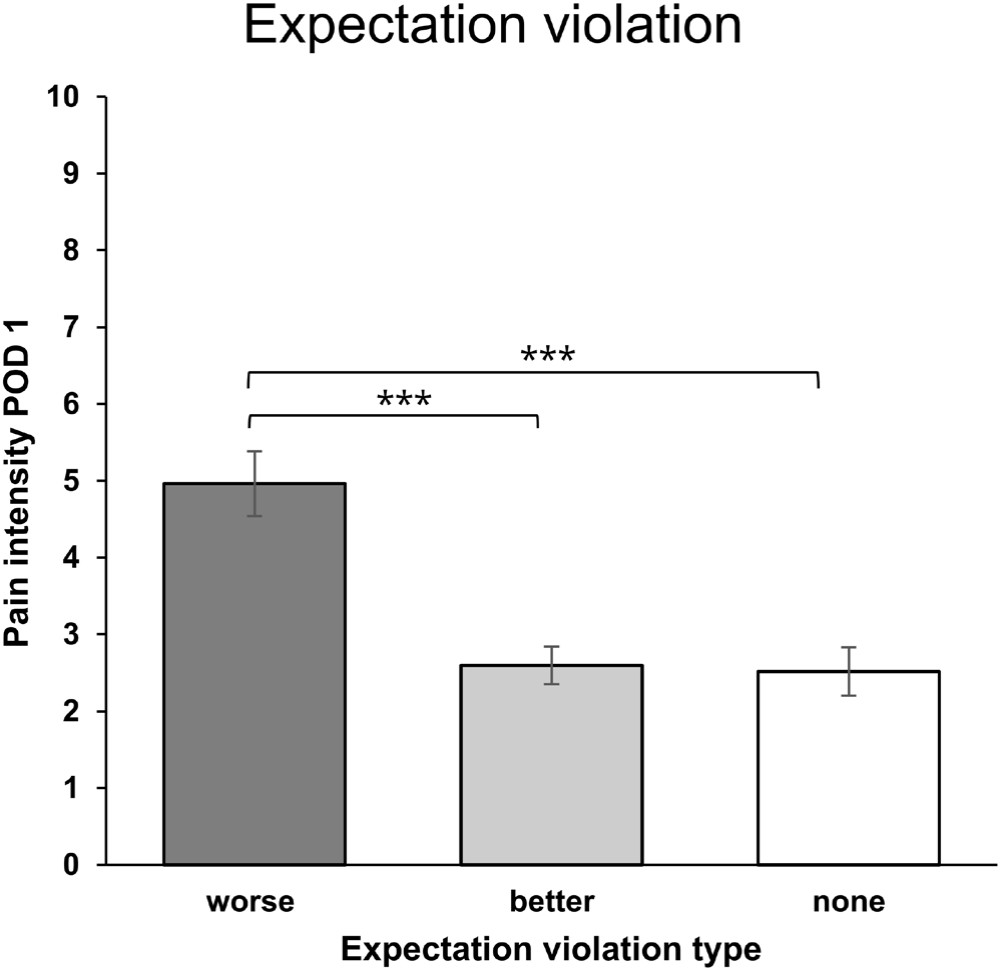
Expectation violation group differences in pain intensity for POD 1. Pain intensity as measured with an 11‐point numerical rating scale on postoperative day 1. ‘worse’ indicates more pain than expected; ‘better’ indicates less pain than expected; ‘none’ indicates a match between preoperative expectations and acute postoperative pain intensity. Error bars represent the standard error of the mean; ***indicates *p* < 0.001.

Regarding postoperative pain intensity for POD 1, feeling worse than expected was reported by *n* = 26 participants (negative expectation violation), feeling better than expected was reported by *n* = 53 participants (positive expectation violation), and no expectation violation was reported by *n* = 33 participants. An independent two‐way analysis of variance was conducted to check for mean differences in pain intensity between expectation violation groups for POD 1. The ANOVA showed a statistically significant main effect for expectation violation type (*F*(2,102.805) = 13.809, *p* < 0.001), with η^2^_p_ = 0.213 indicating a large effect. Neither a main effect of study condition (*p* = 0.698) nor a significant interaction effect was found (*p* = 0.982). Tukey‐corrected post hoc tests showed that POD 1 pain intensity was significantly higher in the negative expectation violation group compared to the positive expectation violation or no expectation violation groups (*M*_Δ_ = 2.306, *t* = 4.862, *p* < 0.001, *d* = 1.195 and *M*_Δ_ = 2.376, *t* = 4.578, *p* < 0.001, *d* = 1.231, respectively, see Figure 4). Pain intensity did not differ between the positive expectation violation group and the no expectation violation group (*p* = 0.986).

**FIGURE 5:**
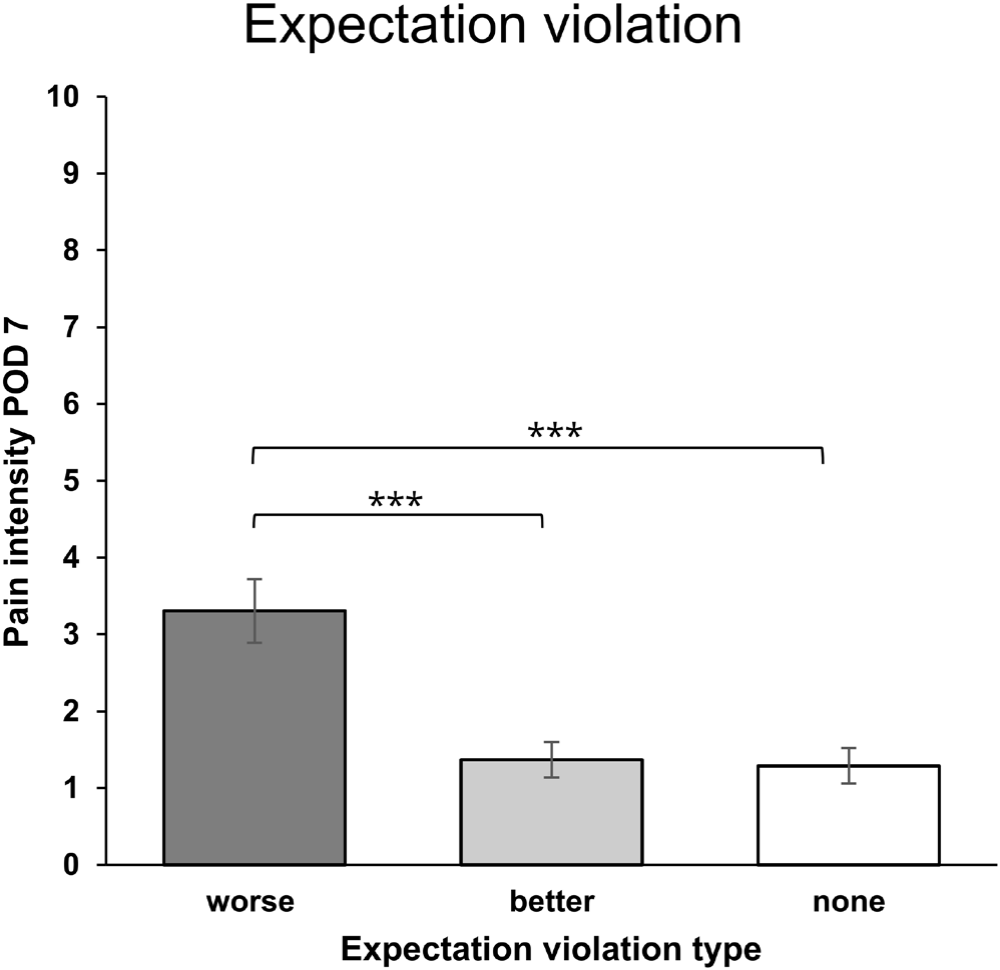
Expectation violation group differences in pain intensity for POD 7. Pain intensity as measured with the 11‐point numerical rating scale on postoperative day 1. ‘worse’ indicates more pain than expected; ‘better’ indicates less pain than expected; ‘none’ indicates a match between preoperative expectations and acute postoperative pain intensity. Error bars represent the standard error of the mean; ***indicates *p* < 0.001.

A similar pattern emerged for POD 7, where feeling worse than expected was reported by *n* = 14 participants, feeling better than expected was reported by *n* = 45 participants, and no expectation violation was reported by *n* = 43. The ANOVA showed a statistically significant main effect for expectation violation type (*F*(2, 47.189) = 10.375, *p* < 0.001), with η^2^_p_ = 0.182 indicating a large effect. Neither a main effect of study condition (*p* = 0.670), nor a significant interaction effect was found (*p* = 0.551). Tukey‐corrected post hoc tests indicated that POD 7 pain intensity was significantly higher in the negative expectation violation group compared to the positive expectation violation or no expectation violation groups (*M*_Δ_ = 1.976, *t* = 4.213, *p* < 0.001, *d* = 1.310 and *M*_Δ_ = 2.041, *t* = 4.333, *p* < 0.001, *d* = 1.353, respectively, see Figure 5). Pain intensity did not differ between the positive expectation violation group and the no expectation violation group (*p* = 0.978).

## 4 Discussion

The present randomised controlled trial investigated the effect of a brief video‐based expectation‐focused psychological intervention on postoperative pain intensity in a sample of adult patients undergoing orthopaedic surgery. Specifically, we wanted to test whether an intervention that would be easy to implement and could be delivered without hospital staff leads to a clinically meaningful effect on pain intensity. While the intervention did lead to a significant increase in patients’ treatment expectations, no effects on pain intensity on postoperative days 1 and 7 were found. Exploratory analyses revealed a large difference in postoperative pain intensity between different expectation violation profiles, which are based on patients' subjective, retrospective reports. Notably, patients who exhibited either a positive expectation violation or no expectation violation at all, did not differ regarding pain intensity. In comparison to those two groups, patients with a negative expectation violation reported significantly more postoperative pain.

### 4.1 Intervention Effects

Our main findings contrast previous evidence suggesting that even brief expectation‐focused interventions in clinical settings are effective (Akroyd et al. 2020), also with regard to postoperative pain intensity as a primary outcome (Benson et al. 2019). Importantly however, although both the interventions of Akroyd et al. (2020) and Benson et al. (2019) were verbally delivered, this was done so in person by hospital staff. Further, the intervention content was tailored to the individual patient (Akroyd et al. 2020) and intervention providers specifically conveyed an optimistic and positive attitude (Benson et al. 2019). It is plausible to assume that an intervention delivered by a ‘real’ therapist, perceived as optimistic, warm and competent (Seewald and Rief 2023) leads to greater effects in clinical outcomes compared to an animated character delivering standardised content in a video sequence. As quantity and quality of provider–patient communication represent a mediating factor of the placebo response on its own (Schedlowski et al. 2015), exclusively relying on expectation optimisation by digitally delivered verbal suggestion might not have been sufficient. However, a different method of delivering the digital intervention, such as through mobile health platforms, might also have been effective. One recent study that used a remotely accessible digital intervention platform, albeit broader in scope, reported reduced postoperative pain in the intervention group (Gordon‐Williams et al. 2021). Using digital intervention platforms with modular components would allow the intervention content to be tailored to patients' individual needs. Lastly, the use of a standardised animated video might also have diminished the perceived credibility of the intervention content, which could have hindered expectation updating (Kube et al. 2022) to a relevant degree.

### 4.2 Expectation Violation

Our exploratory analyses indicated a differential association between the expectation violation type and postoperative pain intensity. While it seems reasonable that patients who thought that they would experience less postoperative pain also indicate higher pain levels, the observation that individuals who reported feeling less pain than expected did not differ from those whose expectations were met regarding pain intensity necessitates a closer look at expectation violation in the context of postoperative pain. Based on our findings, avoiding a negative expectation violation (e.g., experiencing more pain than expected) might be more relevant than achieving a positive expectation violation (e.g., experiencing less pain than expected). Drawing on these findings, caution should be exercised when designing novel expectation‐focused interventions so as not to convey too optimistic expectations. While previous expectation optimisation approaches have tended to focus on optimising positive treatment expectations while also strengthening behavioural control expectations (Rief et al. 2017), addressing patients' negative treatment expectations could be even more important, as too positive expectations might lead to negative expectation violations, which seem most influential in our study. For some patients, it might even be beneficial to reduce too optimistic expectations. Since expectation violation was not manipulated experimentally and groups were formed based on participants' self‐reported data, this finding should be interpreted with caution, as no causal relationship can be inferred.

In general, only a few studies have so far conceptualised and assessed expectation violation as a multifaceted construct which goes beyond having one's expectations fulfilled or not (Mannion et al. 2009). Expectation violation, however, might be a promising construct representing the (mis)match of preoperatively targeted treatment expectations and clinical reality and it might further be influential for patient‐reported outcomes in medical treatments.

### 4.3 Limitations

This study has several strengths, such as its high internal validity due to the randomised controlled trial design, the highly standardised expectation‐focused intervention, and the use of a highly comparable active control condition. There are a number of limitations that need to be considered. First, although we found a positive intervention effect on overall patients’ treatment expectations, the effect sizes were very small and no effect on pain intensity could be detected. The limited magnitude of the expectation change may be partly due to the brevity (less than 10 min in overall duration) and the format (animated, video‐based) of our intervention. As a result, the increase in expectations was likely insufficient to produce clinically meaningful changes in pain intensity. It is imperative for future research to determine the sufficient ‘dose’ of expectation‐focused interventions. Another reason could be that participants were more sensitive to their own expectations due to the explicit discussion of the role of expectations, which could have led to a bias in the processing of the intervention content. Second, patients' baseline treatment expectations were already very positive, limiting the possibility of meaningful expectation optimisation. Future studies could exclude those patients who already have positive treatment expectations. Third, although the randomised controlled trial design enabled rigorous standardisation, unforeseeable surgery cancellations, leading to more than 3 days between intervention delivery and surgery in some cases, might have diminished the presumed effect of our intervention. This variability might have led to a diminished internal validity. Moreover, the assumption that expectations in the SMC group remained constant during the manipulation check may be limited, as a second measurement could have detected potential short‐term changes, even in the absence of an intervention. Further, while we assessed analgesic use between POD1 and POD7, we did not ask participants about the use of nonpharmacological interventions. Additionally, other confounding variables may have influenced the assessment. For example, social support has been shown to be associated with acute postoperative pain (Ai et al. 2024). Regarding the assessment of postoperative pain, the inclusion of an additional outcome capturing the affective dimension of pain (Price 2000), for example, pain unpleasantness, may have strengthened our analysis. Lastly, the assessment of expectation violation might be confounded with memory biases as we asked patients after surgery whether and how their expectations were met. Although ultimately, the perceived degree of violated expectations is highly subjective, future studies should put emphasis on the most valid assessment of expectation violation. An additional, and potentially more objective, measure could involve calculating an expectation violation score that reflects the difference between expected and reported pain intensity.

## 5 Conclusion

In summary, we were able to show that patients’ preoperative treatment expectations predicted postoperative pain. A brief video‐based expectation‐focused intervention leads to optimised treatment expectations in adult patients undergoing elective orthopaedic surgery. However, no effect of our intervention on acute postoperative pain intensity could be demonstrated. Exploratory analyses indicate a differential association between violated expectations and acute postoperative pain. Specifically, patients experiencing a negative expectation violation exhibited more postoperative pain; whereas, interestingly, no difference was found between patients with a positive or no expectation violation. Our findings could inform the development of future expectation‐focused interventions, although a replication of this pattern and an extension to other clinical populations is warranted.

## Author Contributions

Simon Felix Zerth: conceptualisation, methodology, data curation, formal analysis, visualisation, project administration, writing – original draft; Christian Volberg: project administration, supervision, resources, writing – review and editing; Ann‐Kristin Schubert: supervision, writing – review and editing; Vanessa Ketter: resources, writing – review and editing; Monika Sadlonova: supervision, writing – review and editing; Frank Euteneuer: supervision, writing – review and editing; Winfried Rief: supervision, resources, writing – review and editing; Stefan Salzmann: conceptualisation, supervision, writing – review and editing. All authors discussed the results, contributed to the manuscript and approved the final article.

## Disclosure

Plagiarism Declaration**:** We hereby declare that this paper is our own work, except where acknowledged, and has not been submitted elsewhere.

## Conflicts of Interest

W.R. declares to have received honoraria from Boehringer Ingelheim for workshops on Post Covid. The other authors declare no conflicts of interest.

## Supporting information


**Data S1:** ejp70169‐sup‐0001‐supinfo.docx.
