# Supplementary material for: Optimising Treatment Expectations Using a Video‐Based Intervention in Orthopaedic Surgery: Results From a Randomised Controlled Trial
Source: Eur J Pain. 2025 Nov 15;29(10):e70169. doi: 10.1002/ejp.70169 (PMC12619051; doi:10.1002/ejp.70169)
Supplement: Supplementary file 1 — Data S1: ejp70169‐sup‐0001‐supinfo.docx. [file EJP-29-0-s001.docx]

**Optimizing treatment expectations using a video-based intervention in orthopedic surgery: Results from a randomized controlled trial**

**Supplementary material**

**Appendix S1. Translations of intervention and control condition video scripts**

**Intervention script (translation of the original German script)**

Welcome to our video-based information session in the run-up to your planned surgery. Over the next few minutes, we would like to provide you with further information on the subject of surgery in addition to your standard explanation. You will receive in-depth information on surgeries for bone fractures, as well as specific information on psychological factors that can influence the pain intensity after surgery. The aim is to provide you with realistic information about your surgery so that you are as well prepared as possible.

We ask you to watch the video carefully. This will allow you to benefit as much as possible from the content and be optimally prepared for your surgery. Let's get started right away!

Surgical procedures are part of the everyday business of doctors in the fields of surgery and anesthesia. Around 15 million operations are performed in Germany every year. Various types of surgeries on bones and joints, such as the arms and legs, are among the top 20 most frequently performed procedures. Our doctors from the trauma surgery and anesthesia departments are therefore highly experienced, particularly regarding bone fracture surgery.

The risk of surgery following a fracture of the elbow, forearm, hand or leg can generally be described as low. In studies on the side effects of orthopaedic surgery, long-term pain is the most frequently reported side effect. It is important to understand that pain is not a bad thing in itself, but rather a protective function for the body to warn it of a potential injury. So, in essence, it is very useful, even if you don't like feeling it.

Interestingly, psychological and medical research has shown that factors that are only indirectly related to the surgical procedure, the medication, or the medical staff can also have an effect on pain after surgery.

Psychological factors, such as expectations of treatment, pain and the time after surgery, can actually have an impact on pain and health after surgery.

In a systematic review from 2016, researchers were able to demonstrate across 21 studies with a total of 2,600 patients that patients’ treatment expectations have a direct influence on pain intensity and quality of life after surgery.

This effect is demonstrable and not imaginary. The effects of expectations with regard to medical treatment can be measured in the brain and are based on complex psycho-neuro-biological processes. Imaging techniques show, for example, that positive treatment expectations activate various areas of the brain, including the pain-relieving system. Positive expectations can therefore have an influence on how pain is processed and then perceived. An authentic belief in effective treatment alone - in effective pain relief during surgery - can therefore increase the success of the treatment. You can think of it as the body's own pharmacy, which releases messenger substances, for example the body's own pain-relieving opioids.

Conversely, strongly negative expectations and fears regarding pain and side effects are also factors that can influence the experience of pain after surgery. People who are more relaxed and confident about the upcoming procedure have on average less acute pain after the surgery than people who are highly worried and anxious about the procedure.

Finally, we would like to let you know that you are in good hands with us and that you are in one of the most modern anesthesia departments in Germany. Throughout the operation, an anesthetist will always be at your side to ensure that you do not feel any pain. Feeling pain after the surgery can be normal and should not worry you greatly. If you have any questions, please feel free to discuss them with the anesthesia team. If you are in pain, do not hesitate to speak to the clinic staff, who will be able to assist you with pain relief.

Should you feel pain after surgery, other strategies may also help you to increase your well-being and alleviate the pain. These include distraction, for example with your favorite music or a book, but also social support from friends and family.

We would now like to invite you to take part in a short imagination exercise. In our experience, patients report that this is good for them and helps them to form a more positive expectation with regard to the surgery. Position yourself as comfortably as possible in the chair and close your eyes if you wish.

Now briefly think again about what you have seen and heard in this video ... [5sec]

Internalize once again why you are undergoing the procedure and the associated inconvenience: What will you be able to do again in a few weeks? What movements will you be able to perform again? [5sec]

Perhaps you can find an inner image that gives you strength and expresses a realistic and positive attitude.

It could be a picture of you doing a hobby that you are looking forward to again... Or a picture of you in a place where you can relax and look forward to the surgery with calmness [5sec]

Think about it for a moment and then save a positive image in your mind. [5sec]

If you wish, you can recall the image again on the day of the surgery. [5sec]

Now slowly become more aware of your thoughts here in the room and feel free to open your eyes again if they are still closed.

You are welcome to use the time until your procedure to think back to the content of these videos. How do you want to face the upcoming procedure? What can you do for yourself if you experience pain after the procedure despite good anesthesia?

This brings us to the end of our video on additional surgery preparation. We hope you were able to take away some interesting aspects for yourself.

The entire study team from the Department of Anesthesia and Trauma Surgery at Marburg University Hospital and the Clinical Psychology and Psychotherapy working group at Philipps University would like to thank you for participating in our study.

You will now be asked to complete a short questionnaire. You will be contacted again the day after the operation and one week after the operation to answer two short questionnaires. We wish you all the best for the upcoming operation - and have a nice day.

**Control group script (translation of the original German script)**

Welcome to our video-based information session in the run-up to your planned operation. Over the next few minutes, we would like to provide you with additional information on top of your standard information. The focus of the video is on lifestyle and how it can be related to bone healing.

We ask you to watch the video carefully. This will allow you to get the most out of the content. Let's get started right away!

Research over the last few decades has repeatedly emphasized the role of nutrition in bone healing. But what is the current status? That's what this short video is about.

Nutrition, and therefore also the intake of minerals and vitamins, has an influence on our bones.

Calcium, for example, plays a central role in the composition of bones. 99% of the calcium in our body is bound in the skeleton. If there is a deficiency, calcium is released from the bone, which gradually impairs bone strength. Recent scientific findings show that calcium can be absorbed very well by the body through dark green vegetables, nuts, seeds, some fruits and various types of mineral water. Examples include bananas, avocado, almonds, hazelnuts and walnuts.

Another very important mineral is magnesium, because: Calcium only works together with magnesium. A ratio of 2:1 is recommended so that the bones can benefit as much as possible from calcium and magnesium. Good sources of magnesium include oatmeal, salad, wholegrain cereals, lentils and nuts.

In addition to minerals, vitamins are also important for bones. Vitamin D is one of the few vitamins that humans can produce themselves. As a rule, the body develops 80-90% of the vitamin in the skin with the help of sunlight, or more precisely: UV-B radiation. Vitamin D helps the body to absorb the minerals calcium and phosphate from the intestines and incorporate them into the bones. A long-term vitamin D deficiency can contribute to osteoporosis, especially in old age, which reduces the fracture resistance of bones.

Vitamin K is also actively involved in bone formation. Osteocalcin, a specific protein that binds calcium and thus leads to healthy bone formation, is only formed under the influence of vitamin K, for example. Typical sources of vitamin K are also green vegetables such as kale, Brussels sprouts, broccoli and spinach.

And probably the best-known vitamin - vitamin C - should not be neglected when it comes to bones. It is good for the body's own production of collagen and stimulates the bone-building cells. Bones are mainly made up of minerals such as calcium and magnesium. When these and other minerals combine with collagen, the bone is built up and hardens, but without losing its important flexibility.

In summary, a healthy and balanced diet is important for fracture healing. A dairy- and meat-free diet can often contribute better to healing, as it provides the right combination of nutrients and thus improves absorption into the bones. Animal protein in dairy products can contribute to increased calcium excretion, although it is not yet scientifically clear whether increased calcium excretion influences bone resorption.

Too much phosphate, alcohol, caffeine, sugar, salt and fat should be avoided, as they can lead to increased excretion of calcium and magnesium, among other things.

Studies also show that giving up cigarettes in the weeks following the operation improves healing.

We would now like to finish with a short mental exercise. Position yourself as comfortably as possible on the chair and close your eyes if you wish. I will ask you a few questions to which you do not have to answer. It's just a matter of you thinking about them and giving yourself an answer in your mind. [5sec]

Now think back over the last few weeks and consider to what extent what you have just heard is already integrated into your everyday life. [5sec]

Have you been able to enjoy a few vitamin D-rich rays of sunshine? [5sec]

What have you eaten in the last few days? [5sec]

Would you have thought that aspects such as diet and lifestyle can also have an effect on bone healing? [5sec]

Now save an inner image in your mind that matches the content of this video. [2sec] Some patients, for example, choose an inner image of pleasant rays of sunshine. [5sec]

Now slowly return to the room with your thoughts and feel free to open your eyes again if they are still closed. [5sec]

This brings us to the end of our video on additional surgery preparation. We hope you were able to take away some interesting aspects for yourself.

The entire study team from the Department of Anesthesia and Trauma Surgery at Marburg University Hospital and the Clinical Psychology and Psychotherapy working group at Philipps University would like to thank you for participating in our study.

You will now be asked to complete a short questionnaire. You will be contacted again the day after the operation and one week after the operation to answer two short questionnaires. We wish you all the best for the upcoming operation - and have a nice day.

**Appendix S2. Images of both the intervention and control video.**

**
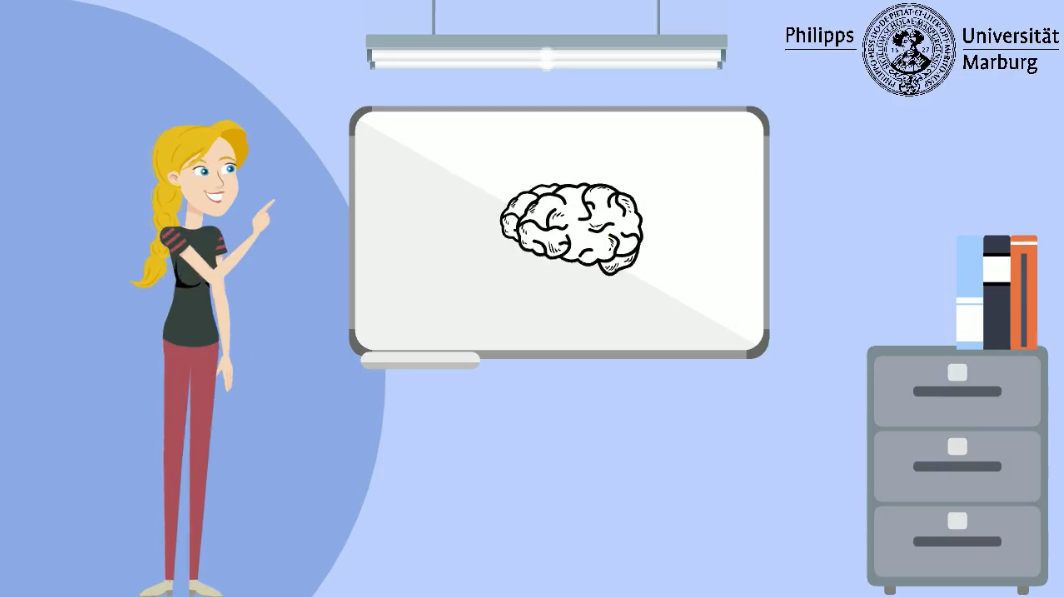
**

**
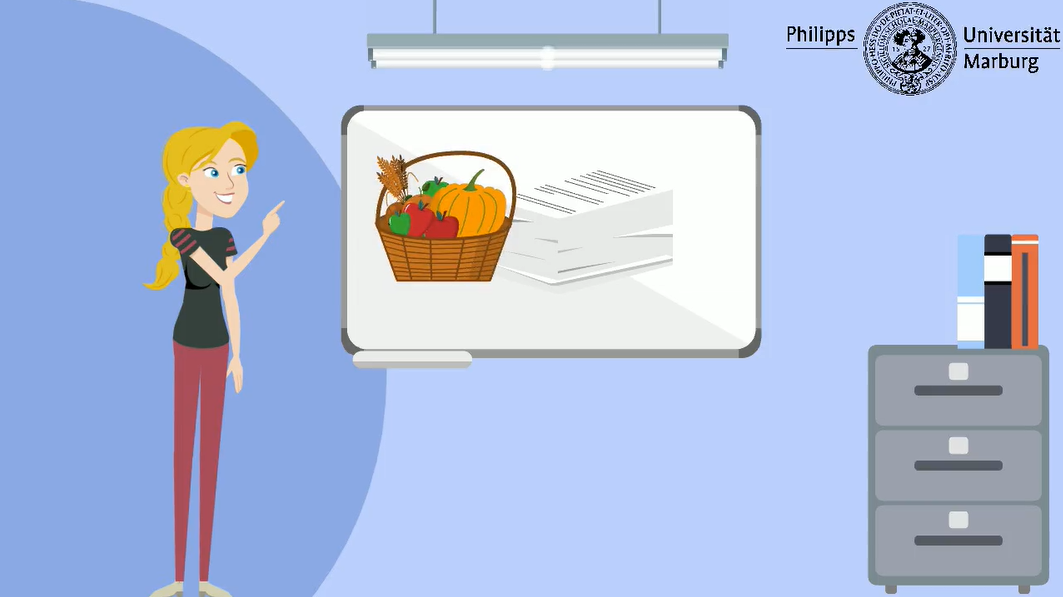
**

**Appendix S3. Additional sensitivity analysis (main analysis with 3 extreme outliers excluded)**

Constrained longitudinal data analysis was used to examine differences in pain intensity from baseline to post- (POD1) and follow-up (POD7) measurements. Prior to this sensitivity analysis, three extreme outliers at POD7 (values > Q3 + 3*IQR, namely IDs 18, 24, and 25) were excluded. The analysis, as described in the section 2.7 of the manuscript, was replicated. The results are as follows:

A significant effect of time was found (*F*(2,104.69) = 73.93, *p*<.001). However, no significant interaction effect between time and treatment group was detected for pain intensity*, F*(4, 101.48) = 1.09, *p* = .367. Adjusted means (± SE) for POD 1 pain intensity were 2.76 ± 0.32 (SMC), 3.35 ± 0.37 (control), and 3.14 ± 0.35 (intervention). Neither the intervention condition nor the active control condition differed from SMC at POD 1 (intervention vs. SMC MΔ = 0.385, 95% CI [−0.549, 1.318], *t*(106.04) = 0.82, *p* = .416; control vs. SMC MΔ = 0.588, 95% CI [−0.374, 1.550]*, t*(106.75) = 1.21, *p* = .228). For POD 7, the adjusted means were 1.56 ± 0.23 (SMC), 1.30 ± 0.24 (control), and 1.59 ± 0.24 (intervention). Again, no between-group differences were detected (intervention vs. SMC MΔ = 0.026, 95% CI [−0.613, 0.666], *t*(99.11) = 0.08, *p* = .935; control vs. SMC MΔ = −0.262, 95% CI [−0.905, 0.382], t(97.54) = −0.81, *p* = .421). Results were essentially unchanged after excluding extreme outliers.
